# *ADCY5* Gene Expression in Adipose Tissue Is Related to Obesity in Men and Mice

**DOI:** 10.1371/journal.pone.0120742

**Published:** 2015-03-20

**Authors:** Anja Knigge, Nora Klöting, Michael R. Schön, Arne Dietrich, Mathias Fasshauer, Daniel Gärtner, Tobias Lohmann, Miriam Dreßler, Michael Stumvoll, Peter Kovacs, Matthias Blüher

**Affiliations:** 1 Department of Medicine, Dermatology und Neurology, Department of Endocrinology und Nephrology, University of Leipzig, Leipzig, Germany; 2 Junior Research Group 2 “Animal models of obesity”, Integriertes Forschungs- und Behandlungszentrum (IFB) Adiposity Diseases, University of Leipzig, Leipzig, Germany; 3 Clinic of Visceral Surgery, Städtisches Klinikum Karlsruhe, Karlsruhe, Germany; 4 Department of Surgery, University of Leipzig, Leipzig, Germany; 5 Municipal Clinic Dresden-Neustadt, Dresden, Germany; 6 Integriertes Forschungs- und Behandlungszentrum (IFB) Adiposity Diseases, University of Leipzig, Leipzig, Germany; GDC, GERMANY

## Abstract

Genome wide association studies revealed an association of the single nucleotide polymorphism rs11708067 within the ADCY5 gene—encoding adenylate cyclase 5—with increased type 2 diabetes (T2D) risk and higher fasting glucose. However, it remains unclear whether the association between ADCY5 variants and glycemic traits may involve adipose tissue (AT) related mechanisms. We therefore tested the hypothesis that *ADCY5* mRNA expression in human and mouse AT is related to obesity, fat distribution, T2D in humans and high fat diet (HFD) in mice. We measured *ADCY5* mRNA expression in paired samples of visceral and subcutaneous adipose tissue from 244 individuals with a wide range of body weight and parameters of hyperglycemia, which have been genotyped for rs11708067. In addition, AT *ADCY5* mRNA was assessed in C57BL/6NTac which underwent a 10 weeks standard chow (n = 6) or high fat diet (HFD, n = 6). In humans, visceral *ADCY5* expression is significantly higher in obese compared to lean individuals. *ADCY5* expression correlates with BMI, body fat mass, circulating leptin, fat distribution, waist and hip circumference, but not with fasting plasma glucose and HbA1c. *Adcy5* expression in mouse AT is significantly higher after a HFD compared to chow (p<0.05). Importantly, rs11708067 is not associated with *ADCY5* mRNA expression levels in either fat depot in any of the genetic models tested. Our results suggest that changes in AT *ADCY5* expression are related to obesity and fat distribution, but not with impaired glucose metabolism and T2D. However, altered *ADCY5* expression in AT does not seem to be the mechanism underlying the association between rs11708067 and increased T2D risk.

## Introduction

In a meta-analysis of genome wide association studies (GWAS) several genetic loci associated with fasting diabetes-related quantitative traits (fasting plasma glucose, fasting plasma insulin, beta-cell function (HOMA-B) and insulin resistance (HOMA-IR) have been identified [1]. Single nucleotide polymorphisms (SNPs) within the ADCY5 gene, which encodes adenylate cyclase 5, are associated with elevated fasting glucose and increased risk to develop type 2 diabetes (T2D) [1]. Recently, it has been shown that *ADCY5* mRNA expression in islets is lower in carriers of high risk alleles at rs11708067 and maybe linked to fasting hyperglycemia and higher T2D risk [2]. Associations between rs11708067 lower birth weight [3] and gestational diabetes has also been shown [4]. Moreover, associations between the major allele (C) of *ADCY5* variant rs2877716 and decreased fasting plasma insulin, peak insulin and increased QUICKI and Matsuda insulin sensitivity index were found in obese children [5].

ADCY5 is a member of the adenylate cyclase family, which consists of twelve domain transmembrane proteins that catalyzes the conversion of ATP to cyclic AMP, the second messenger for G protein coupled receptors [6]. Adenylate cyclases (AC) are ATP-pyrophoshate lyases which convert Adenosine triphosphate (ATP) into the second messenger cyclic adenosine monophosphate (cAMP) and pyrophosphate [6]. cAMP itself plays an important role in glycogen, sugar and lipid metabolism by intracellular signal transduction [6]. ADCY5 has been described to mediate renin release [7], influence heart rate [8] and plays a role in dopamine homeostasis in the central nervous system [9].

Interestingly, ADCY5 has been identified in an exome sequencing study as candidate gene for familial exceptional longevity [10]. In this context, transgenic expression of miR-17 causes ADCY5 silencing which contributes to extended lifespan of mice via promotion of autophagy and repression cellular senescence and apoptosis [11]. Adipose tissue mass, distribution and function are closely related to longevity [12] and the individual risk to develop metabolic diseases including T2D [13]. Despite the robust association of ADCY5 variants and metabolic traits, potential mechanisms underlying the effects of these polymorphic variants at the level of adipose tissue need to be explored.

We therefore tested the hypotheses that (1) *ADCY5* mRNA expression in adipose tissue is related to obesity, fat distribution and T2D in humans, (2) ADCY5 rs11708067 genotype contributes to alterations in *ADCY5* expression and phenotype and (3) high fat feeding of mice results in *ADCY5* expression changes in AT.

## Materials and Methods

### Study participants

Paired samples of visceral (VAT) and subcutaneous (SAT) adipose tissue were obtained from 244 Caucasian men (n = 87) and women (n = 157), who underwent open abdominal surgery for weight reduction surgery (n = 188), abdominal injuries (n = 11), explorative laparotomy (n = 12), cholecystectomy (n = 24) or appendectomy (n = 9) at three medical centers (University of Leipzig, Municipal Clinic Dresden-Neustadt and Municipal Clinic Karlsruhe, all Germany). The mean age of study participants was 51 years (49±15 in women and 53±14 in men) and mean BMI was 42kg/m^2^ (36.2±14.4 in women and 39.7±12.7 in men) (Table 1). All study participants had a stable weight with no fluctuations of more than 2% of the body weight for at least three months before surgery. Patients with severe conditions including generalized inflammation or end stage malignant diseases were excluded from the study. Samples of visceral and subcutaneous adipose tissue were immediately frozen in liquid nitrogen after explantation. Visceral AT samples were obtained from the upper left part of the omentum. The study was approved by the Ethics Committee of the University of Leipzig (Reg. number: 017–12–23012012). All participants gave written informed consent before taking part in the study.

**Table 1:** Anthropometric and metabolic characteristics of participants in the study of adipose tissue *ADCY5* mRNA expression (n = 244).

	Men	Women	T2D	No diabetes	Lean	Obese
N	87	157	80	164	43	175
Age (years)	53±14	49±15	53±12	49±16	48±12.9	51.2±15.7
Body weight (kg)	115±49	108±37	140±39*	111±42	63±10.2***	141.1±31.6
Height (m)	1.78±0.06**	1.64±0.07	1.70±0.1	1.69±0.1	1.70±0.1	1.70±0.1
BMI (kg/m²)	39.7±12.7	36.2±14.4	48±13**	39±13	21.9±2.8***	48.7±9.4
Visceral fat area (cm²)	247±181	254±181	415±151**	182±144	47.1±31***	325±162
SC fat area (cm²)	1040±811	1272±778	1519±611*	1048±825	55±26***	1626±490
CT ratio	0.5±0.7	0.33±0.42	0.48±0.81	0.4±0.4	1.1±0.9**	0.2±0.1
Body fat (%)	34±14	38±10	42±10*	34±12	19±3.5**	42.7±7.2
Waist (cm)	128±35*	119±30	144±27**	111±29	77.9±15***	137.0±23.3
Hip (cm)	129±32	130±29	138±25**	126±31	87±9.7***	144.8±20.3
WHR	0.99±0.1*	0.91±0.1	1.05±0.08*	0.9±0.1	0.89±0.1**	0.95±0.1
Fasting plasma glucose (mmol/l)	5.3±0.6	5.3±0.5	7.7±2.0**	5.3±0.5	5.6±1.1	6.2±1.7

Data are means ± SD.

*p<0.05,

**p<0.01,

***p<0.001 for comparisons between men and women, individuals with T2D versus no diabetes or lean versus obese.

BMI, body mass index, WHR, waist-hip ratio, 2h oGTT, 2 hour oral glucose tolerance test, GIR, glucose infusion rate, hsCRP, high sensitive C-reactive protein, HDL, high-density lipoprotein, LDL, how-density lipoprotein, SC subcutaneous

### Measurement of body fat content, glucose metabolism, insulin sensitivity

BMI was calculated as weight divided by squared height. Hip circumference was measured over the buttocks; waist circumference was measured at the midpoint between the lower ribs and iliac crest. Percentage body fat was measured by dual X-ray absorptiometry (DEXA) and or bioimpedance analysis. Abdominal visceral and subcutaneous fat areas were calculated using computed tomography (CT) or MRI scans at the level of L4–L5 as previously described [14, 15]. Three days before the oral tolerance test (OGTT), patients documented a high-carbohydrate diet in diet protocols. The OGTT was performed after an overnight fast with 75 g standardized glucose solution (Glucodex Solution 75 g; Merieux, Montreal, QC, Canada). Venous blood samples were taken at 0, 60, and 120 min for measurements of plasma glucose concentrations. Insulin sensitivity was assessed using the HOMA-IR index or with the euglycemic-hyperinsulinemic clamp method as described previously [16].

### Analyses of laboratory parameters in blood samples

All baseline blood samples were collected between 8 and 10 am after an overnight fast to determine glucose, insulin and standard laboratory parameters. Plasma insulin was measured with a two-site chemiluminescent enzyme immunometric assay for the IMMULITE automated analyzer (Diagnostic Products Corporation, Los Angeles, CA, USA). Serum high-sensitive CRP, adiponectin, and leptin, were measured as previously described [17].

### Characterization of adipose tissue samples

During surgery, adipose tissue samples were taken from the abdominal SC and the intraabdominal omental fat depots at defined locations. Adipose tissue was analyzed as a whole (immediately frozen in liquid nitrogen after explantation) for histology and mRNA expression analyses. Additional adipose tissue samples were collected in 37°C PBS buffer and adipocytes were isolated by collagenase (1 mg/ml) digestion. Immediately after collagenase digestion, adipocytes were fixed with osmic acid, incubated for 48 hours at 37°C. To determine adipocyte number, 200 μl aliquots of adipocytes were fixed with osmic acid, incubated for 48 hours at 37°C and counted in a Coulter counter (Multisizer III, Beckman Coulter GmbH, Krefeld, Germany). Histologic analyses and measurement of macrophage count in adipose tissue was performed as previously described [17, 18]. In brief, adipose tissue samples were fixed at room temperature in 4% formaldehyde and embedded in paraffin. Five-micrometer sections were mounted on glass slides, deparaffinized in xylol, and stained for CD68 using anti-CD68 monoclonal mouse antihuman antibody (Dako, Glostrup, Denmark; close PGM1 M0876, dilution 1:100). Macrophages were identified in the adipose parenchyma when cytoplasmic staining for CD68 was present along with an identifiable mononuclear nucleus and presented as the number per 100 adipocytes (percent macrophages).

### ADCY5 mRNA expression studies

Human *ADCY5* mRNA expression was measured by quantitative real-time RT-PCR in a fluorescent temperature cycler using the TaqMan Gene Expression Assay Hs00766287_m1 that contains premixed fluorescence labeled probes and TaqMan 2xUniversal PCR Master Mix (Applied Biosystems, Darmstadt, Germany). Total RNA extracted from AT samples was isolated using TRIzol (Life Technologies, Grand Island, NY). 1μg RNA was reverse transcribed using standard reagents (Life Technologies, Grand Island, NY). 2μl of each RT reaction was amplified in a 26 μl PCR by using the Brilliant SYBR Green QPCR Core Reagent Kit from Stratagene (La Jolla, CA, USA according to the manufacturer’s instructions. Samples were incubated in the ABI PRISM 7500 sequence detector for an initial denaturation at 95°C for 10min, followed by 40 PCR cycles, each cycle consisting 95°C for 15s, 60°C for 1min, and 72°C for 1min. Probes were designed using standard Software (Applied Biosystems, Inc., Foster City, CA). Expression of human *ADCY5* mRNA was quantified by using the second derivative maximum method of the TaqMan Software (Applied Biosystems, Darmstadt, Germany) determining the crossing points of individual samples by an algorithm which identifies the first turning point of the fluorescence curve. *ADCY5* mRNA expression was calculated relative to the mRNA expression of *Hypoxanthin-Guanin-Phosphoribosyltransferase 1* (*HPRT1*), determined by a pre-developed TaqMan Assay (Hs01003267_m1; Applied Biosystems). Normalization by endogenous control results in arbitrary units (AU). A detection limit cut off was defined at 36 cycles. Amplification of specific transcripts was confirmed by melting curve profiles (cooling the sample to 68°C and heating slowly to 95°C with measurement of fluorescence) at the end of each PCR. The specificity of the PCR was further verified by subjecting the amplification products to agarose gel electrophoresis.

### Genotyping of the rs11708067 variant

Total genomic DNA from whole human blood samples was isolated using the Quickgene DNA Whole Blood Kit according to the manufacturer’s instructions (Fujifilm, Düsseldorf, Deutschland). The TaqMan SNP Genotyping assay (Applied Biosystems, Foster City, CA, USA) was used in DNA samples to determine the respective genotypic groups. The allelic discrimination reaction was performed according to the manufacturer's instructions on an ABI Prism 7500 sequence detector (assay ID: C_8989067_10; Applied Biosystems, Foster City, CA, USA). Genotype frequencies were in Hardy-Weinberg-equilibrium.

### High fat diet study in C57BL/6NTac mice

This study was carried out in strict accordance with the recommendations in the Guide for the Care and Use of Laboratory Animals of the National Institutes of Health. The protocol was approved by the local authorities of the state of Saxony, Germany (Landesdirektion, Regierungspräsidium Leipzig, Germany; Permit Number: T81/12) as recommended by the responsible local animal ethics review board (Use of Laboratory Animals Ethics Committee of the University of Leipzig; registration number: TVV20/08). All efforts were made to minimize suffering. All mice were housed in pathogen-free facilities in groups of three to four at 22 ± 2°C on a 12-h light/dark cycle. C57BL/6NTac were bred and kept in the Animal Laboratories at the University of Leipzig. Animals had *ad libitum* access to water at all times. Twelve healthy lean male C57BL/6NTac mice were randomized into an arm fed a high-fat diet (HFD) containing 55.2% of calories from fat (C1057, Altromin) (N = 6) or to a standard chow diet (N = 6). After 10 weeks of high fat feeding, animals were sacrificed by an isoflurane overdose according to the ARRIVE Guidelines (S1 Table) and paired samples of inguinal subcutaneous and epigonadal adipose tissue were quickly removed. RNA isolation and quantitative real-time PCR was performed as described above. The PCR was performed using 2 μl of sample from each reverse transcription reaction in a final volume of 26 μl (Brilliant SYBR Green QPCR Core Reagent Kit; Stratagene, La Jolla, CA). The following primer pairs were used: *Adcy5*, 5’- TGGAGATGGGAATGGACATGAT-3’ (sense) and 5’-CACGCGCATGTTCACGTT-3′ (antisense). The mRNA levels were quantified using the second derivative maximum method of the TaqMan software (Applied Biosystems), determining the crossing points of individual samples by an algorithm that identifies the first turning point of the fluorescence curve. Amplification of specific transcripts was confirmed by melting curve profiles (cooling the sample to 68°C and heating slowly to 95°C with measurement of fluorescence) at the end of each PCR. Expression of the normalization gene *hypoxanthine phosphoribosyltransferase (HPRT) 1*, was measured using TaqMan Gene Expression Assays (Mm01545399_m1; Applied Biosystems).

### Statistical analyses

Prior to statistical analysis, non-normally distributed parameters were log-transformed to approximate a normal distribution. Differences in genotype frequencies between the obese or T2D cases and healthy control subjects were compared using logistic regression analyses. Genotype-phenotype association analyses were done under the additive model and the presented *p* values are adjusted for age and sex (and BMI for glucose traits). Results of univariate correlation analysis were described with Pearson`s correlation coefficient. Differences in mRNA expression between VAT and SAT were assessed using paired Student’s *t* test. The analysis of associations with quantitative traits was restricted to non-diabetic subjects to avoid diabetes status or treatment masking potential effects of the variants on these phenotypic traits. Statistical analyses were performed using SPSS version 18.0.1 (SPSS; Inc., Chicago, IL, USA). *p* values <0.05 were considered to be statistically significant.

## Results and Discussion

### Fat depot specific ADCY5 mRNA expression

**Fig 1:**
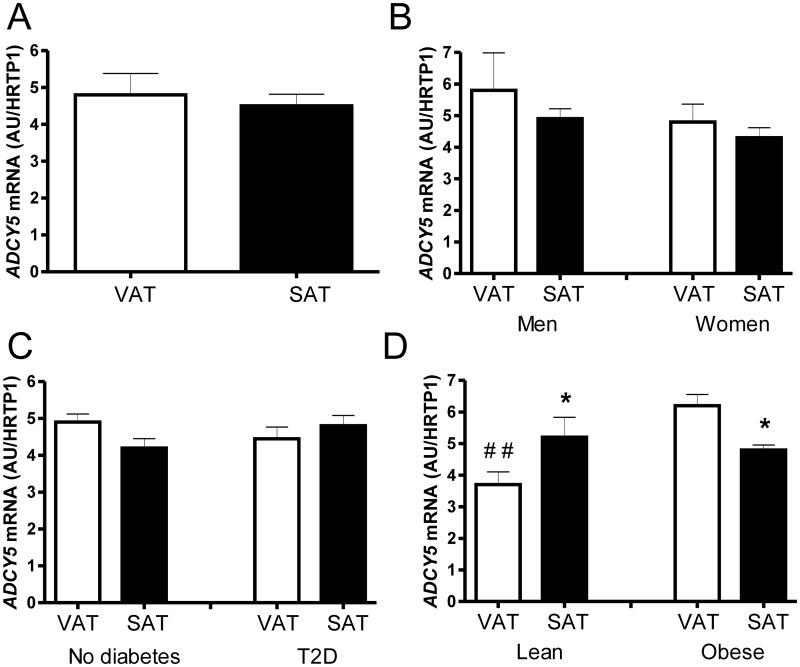
*Adenylate cyclase 5* (*ADCY5*) mRNA expression in human abdominal visceral and subcutaneous adipose tissue. *ADCY5* mRNA expression in adipose tissue (SAT, subcutaneous adipose tissue; VAT, Visceral adipose tissue) from (**A**) the entire study cohort (n = 244), (**B**) 157 women and 87 men, (**C**) individuals with type 2 diabetes (T2D, n = 80) or no diabetes (n = 164), (**D**) study participants with a BMI<25kg/m^2^ (lean, n = 43) or BMI >30kg/m^2^ (obese, n = 175). *ADCY5* mRNA expression was calculated relative to the expression of *HPRT1* (AU = arbitrary units). All data are shown as mean ± SEM. *p<0.05 for comparisons between VAT and SAT *ADCY5* expression; ##p<0.01 for comparisons between lean and obese individuals within each fat depot.

We investigated *ADCY5* mRNA expression in visceral and subcutaneous adipose tissue in 244 individuals (157 women and 87 men), which have been further classified into patients with T2D (n = 80) or normal glucose metabolism (n = 164) (Table 1). In addition, we compared subgroups of lean (BMI <25kg/m^2^, n = 43) and obese (BMI >30kg/m^2^, n = 175) individuals (Table 1). In the analysis of the entire study cohort, we did not find significant differences between VAT and SAT *ADCY5* expression (Fig. 1A). Independently of the fat depot, there were also no differences in *ADCY5* expression between men and women (Fig. 1B) and between patients with or without T2D (Fig. 1C). However, obese adipose tissue donors had significantly higher VAT *ADCY5* expression compared to lean individuals (Fig. 1D). Stratification of the study participants into lean and obese also revealed significant fat depot specific *ADCY5* expression differences with higher expression in VAT compared to SAT in obese and the opposite significant difference in lean individuals (Fig. 1D).

### Correlations of ADCY5 mRNA expression with parameters of obesity, glucose metabolism and insulin sensitivity

*ADCY5* mRNA expression significantly correlates between VAT and SAT independently of age, gender and BMI (Table 2). We found significant correlations of both VAT and SAT *ADCY5* mRNA expression with body weight, BMI, % body fat, waist and hip circumferences, and leptin serum concentrations (Table 2). In addition, VAT *ADCY5* expression correlates with height, abdominal visceral and subcutaneous fat areas, fat distribution (CT ratio) and circulating adiponectin (Table 2). Only for SAT *ADCY5* expression, we found significant relationships with age, serum triglycerides and HDL-cholesterol (Table 2). The correlations between VAT *ADCY5* expression and visceral and subcutaneous fat areas, CT ratio, % body fat, waist and hip circumferences, remained significant even after adjusting for age, gender and BMI, whereas the univariate associations between SAT *ADCY5* expression and body weight, BMI, % body fat, waist and hip circumferences and leptin serum concentrations were independent of age, gender and BMI (Table 2). Importantly, both in VAT and SAT, *ADCY5* expression was not significantly related to maximal (R^2^ = 0.03, p = 0.08) or mean adipocyte size (R^2^ = 0.012, p = 0.13) or macrophage infiltration into adipose tissue (R^2^ = 0.022, p = 0.07). We found the same significant correlations between VAT and SAT *ADCY5* expression after excluding individuals with T2D (data not shown).

**Table 2:** Univariate correlations between *ADCY5* mRNA expression in visceral (VAT) and subcutaneous (SC) adipose tissue and parameters of obesity, insulin sensitivity, and inflammation.

Parameter	SAT *ADCY5* mRNAR^2^; p-value(p-value adjusted for age, gender, BMI)	VAT *ADCY5* mRNAR^2^; p-value(p-value adjusted for age, gender, BMI)
SAT *ADCY5* mRNA	-	**0.09; <0.001 (<0.001)**
VAT *ADCY5* mRNA	**0.09; <0.001 (<0.001)**	-
Age (years)	0.02; 0.03 (n.a.)	0.016; 0.058 (n.a.)
Body weight (kg)	0.02; 0.013 (n.a.)	0.054; <0.001 (n.a.)
Height (m)	0.001; 0.621 (0.723)	0.03; 0.010 (n.a.)
BMI (kg/m^2^)	0.031; 0.008 (n.a.)	0.044; 0.002 (n.a.)
Visceral fat area (cm^2^)	0.011; 0.148 (0.190)	**0.038; 0.007 (0.038)**
SC fat area (cm^2^)	0.009; 0.183 (0.326)	**0.065; <0.001 (0.003)**
CT ratio	0.002; 0.538 (0.988)	**0.048; 0.002 (0.014)**
Body fat (%)	0.051; 0.005 (0.077)	**0.041; 0.013 (0.02)**
Waist (cm)	0.017; 0.047 (0.140)	**0.047; 0.001 (0.01)**
Hip (cm)	0.031; 0.008 (0.071)	**0.061; <0.001 (0.001)**
Fasting plasma glucose (mmol/l)	0.00003; 0.925 (0.866)	0.006; 0.226 (0.24)
HbA1c (%)	0.001; 0.612 (0.914)	0.0006; 0.716 (0.059)
Triglycerides (mmol/l)	0.035; 0.029 (0.142)	0.004; 0.446 (0.097)
HDL-cholesterol (mmol/l)	0.031; 0.044 (0.882)	0.001; 0.255 (0.839)
Adiponectin (μg/ml)	0.007; 0.297 (0.357)	0.018; 0.04 (0.579)
Leptin (pg/ml)	0.025; 0.044 (0.755)	0.038; 0.012 (0.072)

R^2^, Pearson′s correlation coefficient. Analyses were performed only in individuals for which complete data sets were available (n = 240). Significant associations are highlighted in bold if they remained significant after adjusting for age, gender and BMI (in brackets: p-values adjusted for BMI, age, and gender). Significant associations after adjustment are highlighted in bold. (data are log transformed). FPG, fasting plasma glucose; n.a., not applicable.

### Association of rs11708067 with obesity related quantitative traits and ADCY5 mRNA expression

According to the rs11708067 genotype, we found significant differences between AA, AG and GG carriers for fasting plasma glucose and triglycerides (Table 3). In addition, rs11708067 is significantly associated with adiponectin serum concentration (*p* = 0.002, β = 0.587, SE = 0.233, adjusted for age and gender). In contrast to that, we did not find significant associations between rs11708067 and BMI (*p* = 0.336), WHR (*p* = 0.155), HDL-cholesterol (*p* = 0.846), LDL-cholesterol (*p* = 0.255), total cholesterol (*p* = 0.638), triglycerides (*p* = 0.759), fasting plasma insulin (*p* = 0.295), HbA1c (*p* = 0.076), 2hOGTT (*p* = 0.494), leptin serum concentration (*p* = 0.311), creatinine (*p* = 0.758) and % body fat (*p* = 0.989).

**Table 3:** Anthropometric and metabolic characteristics of participants in the study of adipose tissue *ADCY5* mRNA expression according to the rs11708067 genotype.

rs11708067 genotype:	AA	AG	GG
N	150	85	9
SAT *ADCY5* mRNA (AU/HPRT)	3.21±0.5*	4.19±0.7	3.09±1.5
VAT *ADCY5* mRNA (AU/HPRT)	3.55±0.8	3.35±0.7	1.61±1.4^#^ ^,^ ^§^
Age (years)	50±15.7	52±13.5	47±15.0
Body weight (kg)	118.2±45.3	123.5±38.8	117.0±51.2
Height (m)	1.70±0.1	1.70±0.1	1.70±0.1
BMI (kg/m^2^)	41.0±14.6	42.6±12.6	40.3±16.7
Visceral fat area (cm^2^)	259.0±183.1	246.2±176.4	293.1±195.0
SC fat area (cm^2^)	1211.4±805.1	1125.1±798.9	1615.4±398.9
CT ratio	0.4±0.5	0.5±0.7	0.2±0.1
Body fat (%)	36.6±11.9	36.2±11.3	31.0±14.4
Waist (cm)	119.8±33.4	125.2±29.3	122.4±40.7
Hip (cm)	128±21.7	132.9±27.6	128.3±34.5
WHR	0.9±0.1	0.9±0.1	0.9±0.1
Fasting plasma glucose (mmol/l)	5.8±1.2	6.3±1.8*	8.1±3.9^#^ ^,^ ^§^
Fasting plasma insulin (mmol/l)	79.8±109.4	95.2±110.3	69.6±49.2
HbA1c (%)	5.9±1.1	6.0±0.9	6.1±0.6
2h oGTT glucose (mmol/l)	6.7±2.7	7.3±3.1	7.4±0.5
Clamp GIR (μmol/kg/min)	78.6±33.7	73.2±33.3	84.0±40.4
Creatinine (μmol/l)	83.8±51.7	78.5±26.4	95.5±39.2
hsCRP (mg/l)	4.9±1.4	6.1±1.5	3.2±3.5
Triglycerides (mmol/l)	1.5±0.9	1.7±1.0	2.2±1.3^#^ ^,^ ^§^
Cholesterol (mmol/l)	5.0±1.0	5.0±0.9	4.9±1.0
HDL-cholesterol (mmol/l)	1.4±0.4	1.4±0.5	1.4±0.1
LDL-cholesterol (mmol/l)	3.1±1.0	3.3±0.7	3.6±1.7
Adiponectin (μg/ml)	7.6±4.6	7.4±4.5	7.3±7.4
Leptin (pg/ml)	32.8±20.7	39.6±22.6	38.4±28.9

Data are means ± SD.

*p<0.05 for comparisons between AA and AG genotypes,

^#^p<0.05 for comparisons between AA and GG genotypes,

^§^ p<0.05 for comparisons between AG and GG genotypes.

BMI, body mass index, WHR, waist-hip ratio, 2h oGTT, 2 hour oral glucose tolerance test, GIR, glucose infusion rate, hsCRP, high sensitive C-reactive protein, HDL, high-density lipoprotein, LDL, low-density lipoprotein, SC subcutaneous, SAT, subcutaneous adipose tissue, VAT, visceral adipose tissue

Analyses of *ADCY5* mRNA in VAT and SAT identified significant expression differences as a function of the rs11708067 genotype (Table 3). However, rs11708067 is not associated with *ADCY5* mRNA expression levels in either fat depot in any of the genetic models tested (additive, dominant/recessive; all P>0.05 after adjusting for age, sex and BMI). In addition, we performed interaction analyses to test whether there is a statistically plausible interaction between BMI, age, sex and the genotype for *ADCY5* mRNA. Importantly, none of these interaction tests was significant for either subcutaneous or visceral *ADCY5* mRNA as outcome variables (i.e. a total of six tests; all p>0.05).

### High fat feeding increases adipose tissue ADCY5 mRNA expression in mice

**Fig 2:**
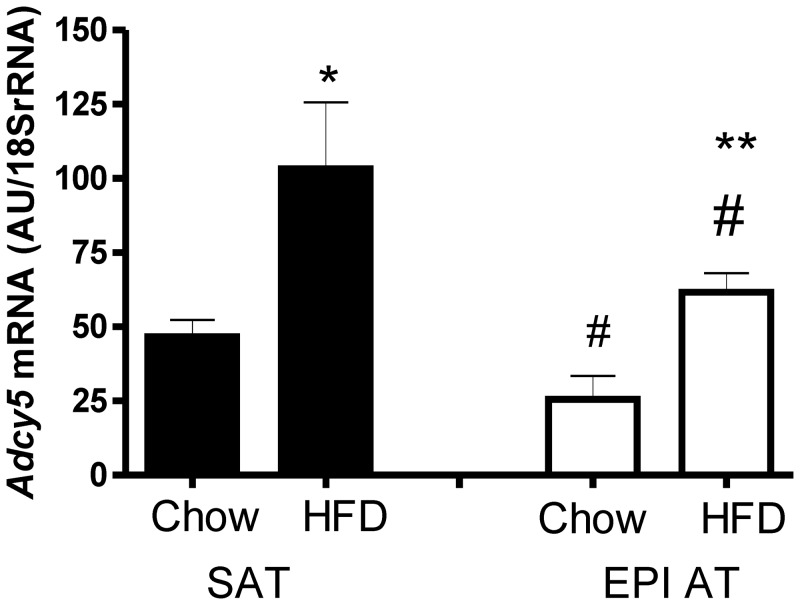
*Adcy5* mRNA expression in adipose tissue of C57BL/6 mice on either chow or high fat diet (HFD). *Adcy5* mRNA expression was calculated relative to the expression of *18S rRNA* (AU = arbitrary units). *p<0.05; **p<0.01 for comparisons between chow and HFD; #p<0.05 for comparisons between fat depots. SAT, subcutaneous adipose tissue; EPI AT, epigonadal adipose tissue.

In C57BL/6NTac, *Adcy5* mRNA expression was detectable in subcutaneous inguinal and epigonadal AT depots with significantly lower expression in epigonadal compared to subcutaneous AT independently of the diet intervention (Fig. 2). Importantly, high fat feeding over 10 weeks resulted in significantly higher *ADCY5* mRNA levels in both AT depots (Fig. 2).

## Discussion

In the current study, we analyzed relationships between *ADCY5* mRNA expression in human visceral and subcutaneous adipose tissue, metabolic traits related to obesity and the ADCY5 genotype at the risk SNP rs11708076. In addition, we tested the hypothesis that *Adcy5* expression in adipose tissue of mice is altered by high fat feeding. We found significantly higher *ADCY5* expression in VAT from obese compared to that of lean individuals. We report here that *ADCY5* expression is strongly related to obesity and fat distribution, because both VAT and SAT *ADCY5* correlate with body weight, BMI, % body fat, waist and hip circumferences, and leptin serum concentrations. These data raise the question whether *ADCY5* expression changes in AT may together with previously reported ADCY5 expression changes at the level of beta cells [2] contribute to obesity related deterioration of glucose metabolism and T2D. However, we did not find significant relationships between *ADCY5* AT expression and parameters of impaired glucose metabolism (fasting plasma glucose and HbA1c). Moreover, patients with T2D did not appear to have higher AT *ADCY5* expression and neither VAT or SAT. Therefore, it is unlikely that the previously reported association between the ADCY5 genotype, higher fasting glucose and increased T2D risk [1] is mediated by *ADCY5* expression changes in AT. Importantly, the cross-sectional design of our study does not allow to draw direct conclusions regarding the causative chain, i.e. whether changes in *ADCY5* mRNA expression precede metabolic alterations or vice versa. The observation that only in obese subjects *ADCY5* mRNA was differentially expressed between SAT and VAT suggests that changes in *ADCY5* expression occur secondary to deterioration of the metabolic profile.

It has been recently reported that SNPs in *ADCY5* (e. g. rs11708076, rs9883204) were associated with T2D [1], decreased pancreatic islet *ADCY5* expression [2], gestational diabetes [4], and lower birth weight [3, 19, 20]. The association with birth weight does not seem to be as robust as the association with T2D, because a recent study [21] could not confirm the birth weight lowering effects of rs9883204, which is in linkage disequilibrium (LD) with rs11708067. A limited sample size and a specific genetic background of the studied Asian population might explain the observed discrepancy [21]. Nevertheless, associations with lower birth weight would support the “fetal insulin hypothesis” by indicating that beta cell function may already be present in pre-natal life [22]. Noteworthy, the ADCY5 risk SNP rs11708067 has been shown to be associated with impaired proinsulin-insulin conversion. [23]. Moreover, Windholz et al. [5] reported associations of the *ADCY5* rs2877716 variant—which is in LD with rs11708067 tested in our work—with parameters of insulin secretion and action (fasting plasma insulin, peak insulin, QUICKI and Matsuda insulin sensitivity index), but not with obesity related traits in obese Caucasian children.

We have to acknowledge, that we could not confirm previously reported associations between rs11708067 and fasting glucose or fasting insulin. This is most likely due to the relatively small sample size (n = 244) and limited statistical power, which may also have been missed in correlation analyses between the ADCY5 genotype and the *ADCY5* mRNA expression in adipose tissue. However, carriers of the GG rs11708067 genotype (n = 9) had significantly lower *ADCY5* VAT expression which supports the notion that this risk genotype also leads to decreased expression in islets [2]. Since this effect was not seen in SAT, the ADCY5 genotype might indirectly contribute to increased T2D risk by promoting visceral fat distribution. Our feeding studies in mice demonstrate that *Adcy5* expression in AT are increasing with high fat diet. These data strongly support the hypothesis that changes in AT *ADCY5* expression may link obesity to T2D. Further studies should investigate whether subsequent changes in adipose tissue morphology (e.g. adipocyte hypertrophy, AT immune cell infiltration) and function are related to *ADCY5* expression and or the ADCY5 genotype. In our study, the sample size was too low to establish such relationships, although non-significant trends could be found for an association between AT *ADCY5* expression, adipocyte size (p = 0.07) and number of macrophages in AT (p = 0.08). Yet, the observed association of rs11708067 with adiponectin serum concentration is at least suggestive for a causal relationship between ADCY5 genotype, *ADCY5* expression and adipose tissue function. Together with the human genotype—*ADCY5* expression data, we postulate that the ADCY5 genotype may modulate the fat depot specific response to high calorie intake.

## Conclusions

Our data do not support the hypothesis that ADCY5 polymorphisms contribute to altered *ADCY5* gene expression in AT. However, independently of the ADCY5 genotype, AT *ADCY5* expression is related to obesity and may contribute to adverse fat distribution and adipose tissue dysfunction. Moreover, our mouse feeding studies suggest that high fat intake may modulate adipose tissue function by increasing expression of *Adcy5*. However, the association between rs11708067, elevated fasting glucose and increased T2D risk detected in genome wide association studies does not seem to be due to *ADCY5* expression changes in adipose tissue.

## Supporting Information

S1 Table Completed ARRIVE Guidelines Checklist. (PDF)
